# Contrasting bioavailability of enterobactin- and ferrichrome-bound iron to SAR11 and other marine heterotrophs

**DOI:** 10.1093/ismeco/ycag113

**Published:** 2026-04-23

**Authors:** Darcy L McRose, Christoph Völker, François M M Morel

**Affiliations:** Department of Civil and Environmental Engineering, Massachusetts Institute of Technology, Cambridge, MA 02139, United States; Marine Biogeosciences Section, Alfred-Wegener-Institut Helmholtz Zentrum für Polar- und Meeresforschung, 27570 Bremerhaven, Germany; Department of Geosciences, Princeton University, Princeton, NJ 08544, United States

**Keywords:** iron, siderophore, SAR11, enterobactin, ferrichrome, secondary metabolite, *Vibrio harveyi*, *Phaeobacter inhibens*

## Abstract

Microbes frequently navigate the environment with the help of small, excreted metabolites. Iron-binding molecules called siderophores are one such set of secondary metabolites that are commonly used by microbes to access the essential trace element iron. Although many marine microbes produce siderophores, a substantial number, including the highly abundant SAR11 clade of *Pelagibacterales*, do not and it has remained unclear whether such nonproducers can access siderophore-bound iron. Here, we show that iron-limited SAR11 cultures fail to grow in the presence of the hydroxamate siderophore ferrichrome but exhibit robust growth in the presence of the catechol siderophore enterobactin. We confirm that this is linked to iron availability using transcriptomic and ^55^Fe radio tracer uptake experiments. This phenotype can be explained by the relative lability of enterobactin-bound iron in seawater, a phenomenon that has been previously observed in field studies and which we demonstrate with a simple kinetic model. Further experiments with the marine heterotrophs *Phaeobacter inhibens* and *Vibrio harveyi* suggest that enterobactin-Fe is unlikely to support the faster growth rates of these organisms without the use of biochemical uptake mechanisms. Overall, our work provides a model of siderophore use that considers bioavailability conferred through both kinetic and biochemical mechanisms and shows that some catechol-bound Fe may be widely available to small, slow growing marine organisms.

## Introduction

Access to the trace metal iron (Fe) limits microbial growth in large swaths of the world’s oceans [1–3]. One microbial response to this limitation is to produce siderophores, small compounds with high affinities for Fe(III) that can help to solubilize and stabilize iron for subsequent cellular uptake [4]. Siderophores are commonly produced by cultured marine heterotrophs [5, 6] but their role in iron bioavailability in the oceans remains incompletely understood. Several decades of research have shown definitively that the majority of iron in the oceans is complexed to organic ligands (see review by [7] and references therein). However, the relative contribution of siderophores to this pool remains an active area of research. Recent advances in mass spectrometry have enabled the detection of siderophores in the waters of the Atlantic, Pacific, and Southern oceans and have started to provide a window into their distributions [8–12]. These measurements of siderophores in seawater are noteworthy beyond the marine context as they represent the most extensive dataset available on the environmental distributions of secondary metabolites of any kind and offer a test of our capacity to establish general trends in small molecule use in natural systems. All major siderophore chemistries—catecholate, hydroxamate, and carboxylate—have been detected, but hydroxamates are by far the most common. Siderophores are typically present at picomolar concentrations and can complex up to 80% of the total Fe pool in marine waters, although this is highly variable spatially and temporally [11–13]. Despite clear demonstrations of their presence in seawater and biosynthesis by marine organisms, siderophore use in the ocean remains somewhat enigmatic: many organisms seem not to produce them, and it is unclear how, if at all, these nonproducing microbes might access siderophore-bound Fe.

The SAR11 clade of *Pelagibacterales*, the most numerically abundant marine heterotrophs [14], is one such group of microbes that appears to have eschewed siderophore production as an iron acquisition strategy. Members of the SAR11 clade possess none of the classical siderophore biosynthetic machinery [15]. Siderophores, by definition, have high affinities for iron, as reflected by experimentally determined stability constants [4] and siderophore-bound Fe is therefore considered inaccessible to organisms without specialized machinery. The best studied strategies for the uptake of siderophore-bound Fe are (i) the reductive mechanism, wherein iron is reduced and liberated from siderophores at the cell surface, or (ii) transport into the cell, using highly specialized transporters, often paired with further intracellular degradation via hydrolases [5, 16–18]. Many bacteria possess transporters for siderophores they do not synthesize themselves and can therefore still access Fe bound to exogenously produced siderophores. This type of “siderophore piracy” is well documented in terrestrial organisms as well as marine heterotrophs [19–22]. However, SAR11 genomes do not encode canonical transporters for siderophores [23, 24], and it has therefore been assumed that this organism is unable to access siderophore-bound Fe of any type.

**Figure 1:**
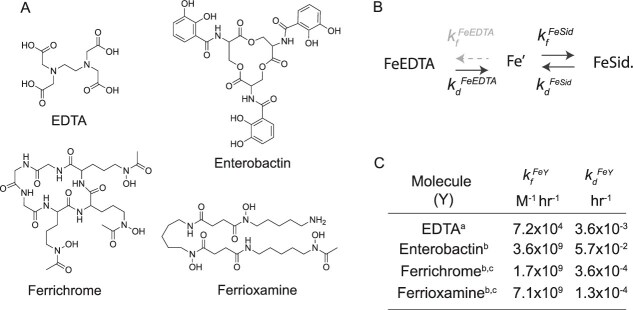
Structures and chemical properties of the iron-binding molecules used in this study. (A) The synthetic iron binder EDTA and the three biological siderophores: enterobactin, ferrichrome, and ferrioxamine. (B) Simple model of the dissociation of FeEDTA to free iron (Fe′) and subsequent complexation by a siderophore. The (re)formation of FeEDTA is neglected due to the presence of high calcium in seawater (see methods). For simplicity, uncomplexed EDTA and siderophores are not shown. (C) Formation (*k_f_^FeY^*) and dissociation (*k_d_^FeY^*) constants for the molecules shown; note that ferrichrome and enterobactin differ by two orders of magnitude. Constants are taken from: a [59], b [33], and c [58]. For enterobactin, note that *k_d_^FeY^* is reported in b [33], but the data used are in [34]. For ferrichrome and ferrioxamine, *k_f_^FeY^* are from b [33] but *k_d_^FeY^* are from c [58]. For ferrichrome and ferrioxamine, dissociation constants were determined using stable isotope exchange. For enterobactin, the siderophore was competed against 1-nitroso-2-naphthol (1N2N).

Yet, our understanding of the extent of siderophore usage in marine systems is continually being revised and expanded. Genes for siderophore uptake have now been found in the genomes of the marine phytoplankton *Synechococcus* and *Prochlorococcus*, organisms that with a few exceptions [25, 26] do not produce siderophores and have long been thought not to utilize them [15, 24]. There has also been growing evidence for marine organisms that can utilize siderophores through nontraditional mechanisms, including endocytosis [27, 28]. These new studies are beginning to offer mechanistic explanations for previous observations of the surprising availability of siderophore-bound iron to marine microbes [29–31]. More than two decades ago, Hutchins *et al.* [29] showed that iron bound to many siderophores was available to both cultured marine phytoplankton and natural phytoplankton populations from the Gulf Stream and Sargasso Sea. The bioavailability of siderophore-bound iron to marine diatoms was also shown in culturing work from Strzepek *et al.* [30] and field studies in the Ross Sea by Kustka *et al.* [31]. While these authors tested many siderophores, an emergent theme was that iron bound to the catechol siderophore enterobactin, widely viewed as the strongest known siderophore due to its very high stability constant [32], was often among the most bioavailable. Consistent with this finding, characterizations of enterobactin-Fe chemistry have shown that in marine waters, enterobactin has a much more moderate conditional stability constant and quite fast dissociation constants ([33, 34], Fig. 1), a feature invoked by Kustka *et al.* [31] as a potential explanation for its seeming bioavailability.

Previous work mostly focused on siderophore availability to large phytoplankton where access may be conferred through a combination of biochemical uptake and the lability of the Fe-enterobactin complex. However, the biggest benefits of enterobactin bioavailability are likely seen by small cells with slow growth rates and low iron requirements, which should enable them to live on siderophore-bound iron without any uptake machinery. We revisited this question by directly challenging cultured representatives of the SAR11 clade with enterobactin as well as the model hydroxamate siderophores ferrichrome and ferrioxamine. Our experiments revealed that SAR11 exhibits robust growth in the presence of enterobactin but fails to grow in the presence of ferrichrome and ferrioxamine. Transcriptomic and ^55^Fe uptake studies confirm the enhanced bioavailability of enterobactin. This result is explained by a simple kinetic model showing that faster dissociation constants of enterobactin compared to ferrichrome and ferrioxamine in seawater allow enterobactin-bound Fe to provide the iron necessary to support the relatively slow growth of SAR11. A second set of experiments with the marine heterotrophs *Vibrio harveyi* and *Phaeobacter inhibens* supports the hypothesis that the faster growth rates seen in these organisms likely require specialized cellular uptake machinery for enterobactin-Fe. Our results provide context for previous work demonstrating the high bioavailability and low iron-binding capacity of enterobactin in seawater and emerging findings that hydroxamate siderophores are far more commonly detected in marine waters than catechols.

## Materials and methods

### SAR11 growth conditions

**Figure 2:**
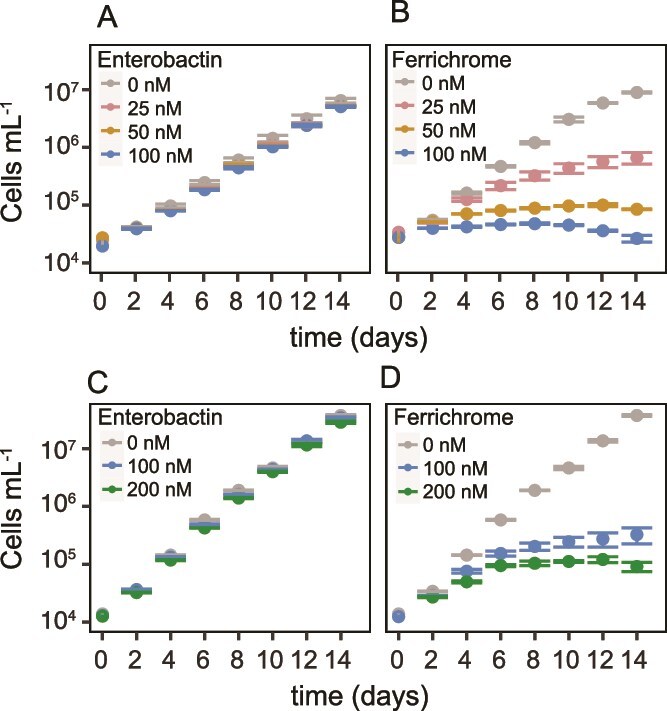
SAR11 shows robust growth in the presence of enterobactin but not ferrichrome. Response of SAR11 HTCC1062 (A, B) and SAR11 HTCC7211 (C, D) to varying amounts of the siderophores enterobactin and ferrichrome. Data shown are the average of biological triplicates (A, B) or duplicates (C, D) ± SD. For panels A and B, 0 nM ferrichrome and 0 nM enterobactin data are from distinct growth experiments. For panels C and D, the 0 nM treatment was only conducted once, and the data are reproduced in both graphs. See Fig. S1 for data from replicate experiments with HTCC1062 and Table S1 for calculated growth rates and yields.

SAR11 HTCC1062 and HTC7211 were gifts from the Giovannoni lab (Oregon State) and Chisholm lab (MIT), respectively. Cultures were grown in polystyrene tissue culture flasks and maintained at 20°C (HTCC1062) or 22°C (HTCC7211) in the dark without shaking in a modified version of AMS1 [35]. Aquil trace metals [36] containing 100 μM ethylenediaminetetraacetic acid (EDTA) without added Fe were used instead of AMS1 trace metals. Measurements of representative media batches by Inductively-Coupled Plasma Mass Spectrometry (ICP-MS) showed background iron was 50–100 nM. Growth medium was prepared in acid cleaned plasticware and sterilized using filtration rather than autoclaving, CO_2_ and air sparging steps were also omitted (this modified version of AMS1 is previously reported in [37]). Before the start of experiments, cells were grown to mid-exponential phase (~1 × 10^6^ cells ml^−1^) and inoculated into experimental treatments. Enterobactin, ferrichrome, and ferrioxamine B were purchased from Sigma or Cayman Chemical and maintained as 10 mM stocks in 9:1 acetonitrile:water (enterobactin) or water (ferrichrome, ferrioxamine B) and kept at −20°C. For initial experiments with SAR11 (Figs 2, S1), 100 μM siderophore stocks were made in water and used to initiate experiments. To avoid degradation, siderophores were added immediately before cells were added (no pre-equilibration). Vehicle controls for acetonitrile were not conducted in these experiments due to the extremely low carryover (100 000× dilution). Due to heightened concern over enterobactin degradation in aqueous solutions, for later experiments (Figs. 4E, 5A and S3), 1 mM enterobactin stocks were maintained in 9:1 acetonitrile:water and added directly to culture media. Due to the larger and continued acetonitrile additions, vehicle controls were conducted for all treatments (i.e. at each time point, all treatments received either enterobactin in 9:1 acetontrile:water or just 9:1 acetontrile:water). Enterobactin and vehicle controls were adjusted at each time point for the volume lost to sampling for flow cytometry (500 μl lost per time point). Our data suggest that acetonitrile has little effect on SAR11 at these concentrations. Cell concentrations were determined by flow cytometry: 500 μl of culture was fixed with glutaraldehyde (1% final) and stored at −80°C. For analysis, samples were thawed and stained with SYBR green nucleic acid stain for 45 min (Fisher, S7567, used at manufacturer’s recommended 1X final concentration) before being analyzed using an Accuri C6 flow cytometer (BD, Figs 2A and B, S1) or Guava easyCyte HT (Figs 2C and D, 4E, 5A, S3).

### Short-term siderophore exposure, RNA extraction, and RT-qPCR

For short-term siderophore exposure experiments, cultures were inoculated at 8 × 10^6^ cells ml^−1^ in media containing either 100 nM enterobactin or 100 nM ferrichrome and incubated in the dark at 22°C. At the start of experiments, 170 ml of culture from untreated controls was harvested by filtration onto 0.1 μm 47 mm polycarbonate filters, flash frozen, and stored at −80°C for later analysis. These “T0” samples were used as the calibrators for gene expression data. After 48 h, 170 ml of culture was harvested from all treatments by filtration as described above, flash frozen, and stored at −80°C for later analysis. RNA isolation was performed with the Qiagen RNEasy kit using lysozyme and proteinase K. Extractions were conducted according to the manufacturer’s instructions except that the lysis buffer was increased to 0.5 ml per sample to ensure full coverage of filters, which were placed directly into the buffer, and an additional 5-min sonication step was added to increase yields. Following isolation, RNA was treated with TURBO DNAse (Invitrogen) using the rigorous protocol (2 μl enzyme reaction^−1^). cDNA synthesis was performed using the iScript cDNA synthesis kit (Bio-Rad) using 500 ng RNA per reaction. Reverse-transcription quantitative polymerase chain reaction (RT-qPCR) experiments were performed using the iScript SYBR green master mix and a CFX duet qPCR machine (Bio-Rad) with the following program: 95°C for 3 min followed by 40 cycles of 95°C for 10 s, 57°C for 30 s. To confirm that qPCR primers amplified the correct gene, amplicons were separately PCR amplified, and Sanger sequenced (see Table S2 for primer sequences). Minus reverse transcriptase (−RT) reactions were performed for all genes and treatments. The lowest amplification detected in minus RTs was at 27 cycles, which was greater than the threshold cycle for any of the genes analyzed. Fold changes were calculated using the delta delta C_T_ method [38] with *rpoD* as the housekeeping gene and the initial time point as the calibrator.

### ^55^Fe uptake experiments

For iron uptake experiments, SAR11 HTCC1062 was grown to mid-exponential phase in iron-free media with 100 μM EDTA. Cells were then concentrated via gentle filtration, washed, and inoculated at a density of 1 × 10^8^ cells ml^−1^ into media containing either 1 μM enterobactin or 1 μM ferrichrome (without EDTA) and 20 nM of the radio-isotope ^55^Fe (as ^55^FeCl_3_, Perkin Elmer), which was pre-equilibrated with siderophores for at least 24 h. Experiments commenced with the addition of cells to media treatments. At each time point (0, 15, 40, and 60 min for Experiment 1, or 0, 10, 40, and 60 min for Experiment 2), 5 ml of cells were filtered onto 0.1 μm polycarbonate filters, washed twice with oxalate EDTA and NaCl [39] and transferred to scintillation cocktail (Ultima Gold, PerkinElmer). A counting efficiency of 30% and a total iron concentration of 120 nM (labelled = 20 nM and background = 100 nM) were used to calculate iron per cell; cell numbers were assumed to be constant over the short experiment. Uptake rates were calculated as the linear regression of Fe per cell vs time for two biological replicates across two separate experiments (four replicates in total).

### Abiotic degradation and liquid chromatography–mass spectrometry

For abiotic degradation experiments, enterobactin was incubated in milliQ water buffered with 1 mM HEPES at pH 8.1. To facilitate easy detection by liquid chromatography–mass spectrometry (LC–MS), the siderophore was added at a final concentration of 20 μM. This experimental setup allowed for direct LC–MS injection without the need for purification and concentration via solid phase extraction, which could introduce further artifacts. Iron (as FeCl_3_) was added at a final concentration of 40 μM. Samples were incubated in 15 ml polystyrene conical tubes in the dark at 22°C and sampled every other day. Samples were immediately frozen at −20°C for LC–MS analysis. Siderophores were detected on an Agilent 1260 liquid chromatography system with a single quadrupole mass spectrometer (Agilent MSD) using a C18 column (Poroshell 120-EC, 50 mm length, 3 mm diameter, 2.7 μm particle size) under a gradient of acidified (0.1% formic acid) water to acidified (0.1% formic acid) acetonitrile over 12 min. Enterobactin was detected using single ion monitoring, in negative mode (*m*/*z* = 668.5), and identity was confirmed by comparison to a standard.

### *Phaeobacter inhibens* and *Vibrio harveyi* growth conditions

*Phaeobacter inhibens* DSM17395 and *V. harveyi* BB120 were cultured using H-Aquil, a defined medium for trace metal studies in marine heterotrophs [37]. For *Phaeobacter* experiments, glucose (10 g l^−1^) was used as the carbon source instead of glycerol. To initiate experiments, cultures were struck onto L-Marine or marine broth plates and grown overnight at 25°C. Single colonies were then inoculated into defined H-Aquil medium without added iron and grown in polystyrene tissue culture flasks with shaking at 200 RPM. To exhaust any cellular iron reserves, a total of three transfers (1:100 dilutions every ~24 h) were conducted before the start of experiments. Growth experiments were conducted in 96-well plates, and growth was monitored using absorbance at 500 nm, read once per hour in a Synergy Epoch-2 Plate reader (Agilent). For aged media experiments, growth medium was maintained in 15 ml polystyrene conical tubes at 22°C in the dark for 14 days before being used for experiments.

### Kinetic model

Ignoring the uptake by the cells, the evolution of Fe${}^{\prime }$ can be calculated by integrating the differential equation below:


(1) \begin{equation*} \frac{d\left[F{e}^{\prime}\right]}{dt}={k}_d^{FeEDTA}\left[ FeEDTA\right]-{k}_f^{FeY}\left[Y\right]\left[F{e}^{\prime}\right]+{k}_d^{FeY}\left[ FeY\right] \end{equation*}

where FeEDTA is the FeEDTA complex (taken to be initially 100 nM), Y represents the free siderophore (enterobactin or ferrichrome), Fe′ is the unbound iron, ${k}_d^{FeEDTA}$ is the dissociation constant for the FeEDTA complex, and ${k}_f^{FeY}$ and ${k}_d^{FeY}$ are the formation and dissociation constants for the Fe-siderophore complex, respectively (following values shown in Fig. 1C). This equation is complemented by a corresponding differential equation for the time evolution of FeY:


(2) \begin{equation*} \frac{d\left[ FeY\right]}{dt}={k}_f^{FeY}\left[Y\right]\left[F{e}^{\prime}\right]-{k}_d^{FeY}\left[ FeY\right] \end{equation*}

As long as the total added ligand is conserved, the free ligand concentration can be calculated from $\left[Y\right]=\left[{Y}_{added}\right]-\left[ FeY\right]$. In the case of enterobactin, however, when we also investigated the consequences of a degradation of apo-enterobactin, the following differential equation has to be solved in addition:


(3) \begin{equation*} \frac{d\left[Y\right]}{dt}=-{k}_f^{FeY}\left[Y\right]\left[F{e}^{\prime}\right]+{k}_d^{FeY}\left[ FeY\right]-{k}_{deg}^Y\left[Y\right] \end{equation*}

where ${k}_{deg}^Y$ is the first-order degradation rate of the apo-form of the ligand. Finally, to a first approximation, one may take the concentration of FeEDTA as constant; a somewhat more accurate solution is obtained by solving a fourth equation for the rate of change of the FeEDTA complex:


(4) \begin{equation*} \frac{d\left[ FeEDTA\right]}{dt}=-{k}_d^{FeEDTA}\left[ FeEDTA\right] \end{equation*}

The model equations (1) to (4) were integrated in time with the solve_idp function from the scientific Python package SciPy, using an implicit variable-order scheme from [40] and automatic stepsize control. Given that concentrations in molar units are on the order of 10^−9^, fairly strict tolerances had to be used for the stepsize control. The initial conditions at  $t=0$ chosen in the integration were:


(5) \begin{equation*} \left(\left[F{e}^{\prime}\right],\left[ FeY\right],\left[ FeEDTA\right],\left[Y\right]\right)=\left(0,0, FeEDTA_{0}, Y_{added}\right) \end{equation*}

where FeEDTA_0_ and Y_added_ are the initial concentrations of FeEDTA (100 nM) and added ligand Y (25/50/100 nM or 1 μM).

### Biological uptake calculation

The cumulative Fe′ uptake by SAR11 for unlimited growth in the growth experiments can be estimated from the initial cell density ${C}_o$, the maximum growth rate obtained during exponential growth in the nonlimited conditions ${\mu}_{max}$, and the cellular uptake rate estimated in the short-term ^55^Fe uptake experiments ${a}_{cell}$. The instantaneous iron uptake calculated from these numbers is


(6) \begin{equation*} {BFe}_{inst}={a}_{cell}{C}_{0\kern0.5em }{e}^{\mu_{max}t} \end{equation*}

Integrating this over time gives the cumulative iron uptake in the experiments, assuming unlimited growth:


(7) \begin{equation*} {BFe}_{cum}=\frac{a_{cell}}{\mu_{max}}\kern0.5em {C}_{0\kern0.5em }{e}^{\mu_{max}t} \end{equation*}

Comparing this calculated Fe′ demand to the abiotically calculated Fe′ from the kinetic model is a simplification for two reasons: First, in the case of iron-limited growth, the iron uptake would be lower than what is calculated here. For this reason, we show the uptake as a wedge, rather than a line in Fig. 4A–D. Second, the uptake of Fe′ by the bacteria would affect the Fe′-siderophore equilibrium and lead to an additional release of Fe′ from the FeY complex. Taking this fully into account, however, requires a model that links the change of Fe′ uptake to changing cell numbers explicitly. This requires information on how SAR11 growth is reduced under iron limitation using a cellular half-saturation constant for iron uptake. To our knowledge, these constants have not been reported for SAR11; we have therefore refrained from making this calculation.

### Statistical analysis

Statistics were performed either in R [41] (ANOVA) or Excel (*t*-tests).

## Results and discussion

### SAR11 grows robustly in the presence of the catechol siderophore enterobactin but not the hydroxamate siderophore ferrichrome

To test the effects of siderophores on the growth of *Pelagibacter ubique*, we established a moderately low iron system (Fe_T_ < 100 nM) that utilizes the synthetic chelator EDTA (100 μM) in combination with either enterobactin or ferrichrome. We next determined growth in response to a gradient of siderophore additions ranging from 25 nM to 100 nM. The presence of as little as 25 nM ferrichrome led to a measured decrease in SAR11 HTCC1062 growth (with growth rates during the exponential phase dropping from 0.54 to 0.36 d^−1^ and yields dropping to ~7% of untreated controls, Fig. 2, Table S1) and 100 nM led to near cessation of growth, consistent with previous observations [42]. In contrast, up to 100 nM enterobactin had much milder effects, growth rates were maintained at ~88%, and yields remained at ~80% of untreated controls regardless of siderophore concentration (Fig. 2, Table S1). In two replicate experiments, we observed similar trends (Fig. S1), albeit with varying effects on cell yield (Table S1). We attribute this variation to fluctuations in levels of background iron, which may be well below 100 nM in some experiments. To further explore this phenotype, we extended our studies to SAR11 HTCC7211, another clade Ia member of this group, observing highly similar results, with clear growth cessation in the presence of 100 nM or 200 nM ferrichrome and almost no effect of enterobactin at either concentration (Fig. 2C and D).

### SAR11 upregulates the iron transport genes *sfuA* and *sfuB* in the presence of ferrichrome but not enterobactin

**Figure 3:**
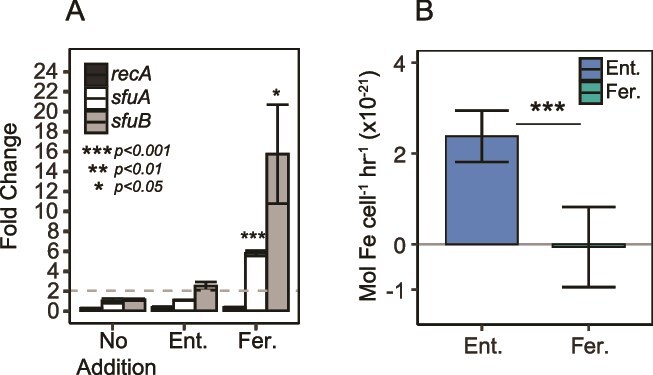
SAR11 access to iron is limited in the presence of ferrichrome but not enterobactin. (A) SAR11 HTCC7211 upregulates iron-stress genes in response to ferrichrome but not enterobactin. Expression of the iron transporters *sfuA* and *sfuB* in response to a 48-h treatment with 100 nM enterobactin, 100 nM ferrichrome, or no addition. The DNA repair gene *recA* is included as a control. Expression is normalized to the no treatment controls at time zero using the sigma factor *rpoD* as the housekeeping gene. Data shown are for biological duplicates ± SD. Statistics shown are from a one-way ANOVA with Tukey post hoc correction and denote differences between the control (no addition) and the indicated treatment for each gene. (B) SAR11 HTCC1062 can access enterobactin- but not ferrichrome-bound Fe. Uptake experiments were conducted using 1 μM siderophore without EDTA. Data shown are for biological quadruplicates conducted across two separate experiments ± SD (see methods). Statistics reflect a two-tailed *t*-test. Ent.: enterobactin, Fer.: ferrichrome.

To ensure that the difference between ferrichrome- and enterobactin-treated cells is truly due to iron as opposed to toxicity or some other mechanism, we examined the expression of genes in HTCC7211 known to be upregulated in response to iron limitation. Previous studies of iron limitation in *P. ubique* HTCC1062 [42] have reported strong upregulation of an iron ABC transporter encoded by *sfuA,* as well as an ATPase and a permease (*sfuB*) and a periplasmic iron binding protein (*sfuC*). Despite high levels of expression for *sfuC* observed previously, homology between HTCC1062 and HTCC7211 was weaker for this gene than for the others, so we chose to focus our qPCR studies on *sfuA* and *sfuB*. To avoid the potentially confounding effects of large growth differences between enterobactin- and ferrichrome-treated cultures (Fig. 2), we examined the short-term response of HTCC7211 to the presence of each siderophore. The expression of *sfuA* and *sfuB* was quantified in cells grown to mid-exponential phase and resuspended at high concentrations (~8 × 10^6^ cells ml^−1^) in media supplemented with either 100 nM ferrichrome, 100 nM enterobactin, or no addition. In the presence of ferrichrome, our results clearly show upregulation of *sfuA* and *sfuB* compared to the initial time point. However, in the presence of either enterobactin or no addition, these iron stress genes show little change (Fig. 3A). It is notable that *sfuB* showed a slight increase in enterobactin treatments, suggesting that the cells may experience mild limitation. As a control, we also examined changes in the expression of *recA*, a gene involved in DNA repair, which showed no change across treatments, confirming that the observed response is driven by iron limitation.

### ^55^Fe uptake rates are higher for enterobactin- vs ferrichrome-bound iron

As a further test of the differences in bioavailability between enterobactin and ferrichrome, we compared ^55^Fe-uptake rates in SAR11 cultures (HTCC1062) in the presence of 1 μM enterobactin or 1 μM ferrichrome. We observed clear differences between the treatments: uptake in the presence of enterobactin was ~2.4 × 10^−21^ mol Fe cell^−1^ h^−1^, whereas uptake rates in the presence of ferrichrome were negligible and rate calculations often yielded negative slopes, reflecting a lack of uptake over the experimental period (Fig. 3B). This result supports our hypothesis that Fe derived from enterobactin is more bioavailable than ferrichrome. To our knowledge, these data represent the first reported iron uptake rates of any kind for SAR11, and when normalized to surface area, our results are remarkably consistent with previous observations of iron uptake by other marine microbes. Lis *et al.* [43] compared the uptake of iron bound to the siderophore ferrioxamine across species by normalizing iron uptake rates (mol cell h^−1^) by siderophore-bound iron (mol l^−1^) and found rates of ~1 × 10^−13^ to 1 × 10^−14^ l cell^−1^ h^−1^ for species with surface areas of <1 μm^2^, the size expected for SAR11 [44, 45]. When normalized similarly, our uptake rates are ~2 × 10^−14^ l cell^−1^ h^−1^ for enterobactin, suggesting that iron uptake in this organism scales predictably with its surface area. Overall, these data provide direct evidence of differences in bioavailability between enterobactin and ferrichrome-bound iron to SAR11.

### Differences in siderophore-Fe chemistries explain SAR11 growth in the presence of enterobactin

The robust growth and iron uptake of SAR11 in the presence of enterobactin may seem curious given the absence of any canonical genes coding for the uptake of this siderophore in the genome [23]. Our results are most simply explained by large differences in the kinetics of Fe binding and release between enterobactin and ferrichrome in seawater (Fig. 1C) shown in a simple model of Fe kinetics in our various treatments that incorporates seawater-specific parameters. As depicted in Fig. 1B, Fe is delivered to the cells from dissociation of FeEDTA, with the resulting unchelated iron (Fe′) equilibrating with a siderophore, namely the catecholate enterobactin or the hydroxamate ferrichrome in the experiments of Fig. 2. In seawater, the free EDTA produced by the dissociation of FeEDTA becomes rapidly bound to Ca^2+^ and plays no further role in the experiments [46]. Although the Fe complexes of enterobactin and ferrichrome have very high proton-independent stability constants in freshwater systems (log *K*_Fe_ *=* 59 and 29, respectively [4]), their effective Fe affinities are much lower in seawater owing principally to their binding to Ca^2+^ and Mg^2+^ (log *K*_Fe_^sw^ *=* 10.8 and 12.9 [33, 34]). Most importantly, in seawater, the rate of dissociation of Fe-enterobactin is much faster than that of Fe-ferrichrome (Fig. 1C).

**Figure 4:**
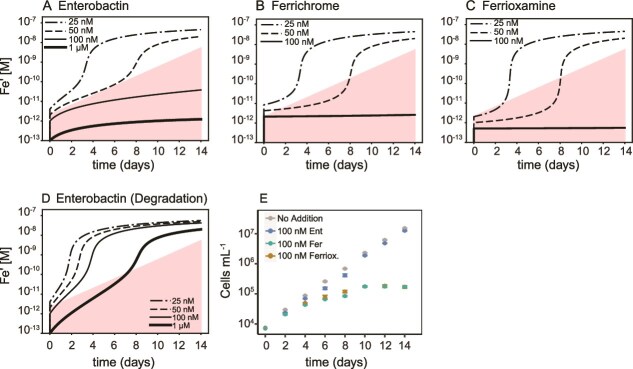
Differences in siderophore kinetics explain growth results. A simple model of free iron (Fe′) supply based on dissociation from FeEDTA and equilibration with enterobactin (A) ferrichrome (B) or ferrioxamine (C) predicts the greatest availability in the presence of enterobactin. The model assumes total iron = FeEDTA initial = 100 nM. D. Fe′ assuming degradation of apo-enterobactin at a rate of 0.58 day^−1^. For (A)–(D), a simple calculation of cellular demand is included assuming a growth rate of 0.55 d^−1^ and uptake of 2.4 × 10^−21^ mol Fe cell^−1^ h^−1^. E. Growth of SAR11 HTCC7211 in the presence of the three siderophores follows kinetic data from (A), (B), and (C). Ent, enterobactin; Fer, ferrichrome; Ferriox., ferrioxamine. See Fig. S2 for abiotic enterobactin degradation data, Table S1 for calculated growth rates and yields, and methods for model details. Data in (E) are the average of biological duplicates ± SD.

The evolution in time of free iron (Fe′) resulting from these reactions can be calculated from a set of differential equations for the rate of change of Fe′, FeEDTA, the iron-siderophore complex FeY, and the apo-form of the siderophore Y (details on the kinetic model are described in the methods section). Solving this over the course of 14 days shows the clear increase in Fe′ over time when enterobactin constants are used, even in the presence of 100 nM enterobactin. In contrast, simulations using ferrichrome maintain low Fe′ in both 50 and 100 nM treatments for the duration of the experiment (Fig. 4A and B). To expand our results, we conducted the same exercise with ferrioxamine, a hydroxamate siderophore with a conditional stability constant (log *K*_Fe_^sw^ *=* 12.1 [33]) and forward and reverse reaction rates comparable to ferrichrome (Fig. 1C). As expected, a kinetic model of Fe′ showed behavior similar to ferrichrome. When grown in the presence of ferrioxamine, SAR11 cell counts largely followed the ferrichrome data (Fig. 4E), consistent with the differences in siderophore chemistries. The available Fe′ can also be compared to a very simple estimate of cellular demand, assuming uptake rates of 2.4 × 10^−21^ mol Fe cell^−1^ h^−1^ (Fig. 3B) and a constant growth rate of 0.55 day^−1^ (Table S1). This calculation shows that Fe supply is sufficient to support growth in the presence of 25 and 50 nM enterobactin but slightly below demand at 100 nM. In contrast, models of ferrichrome and ferrioxamine suggest that only the 25 nM treatment provides sufficient iron.

**Figure 5:**
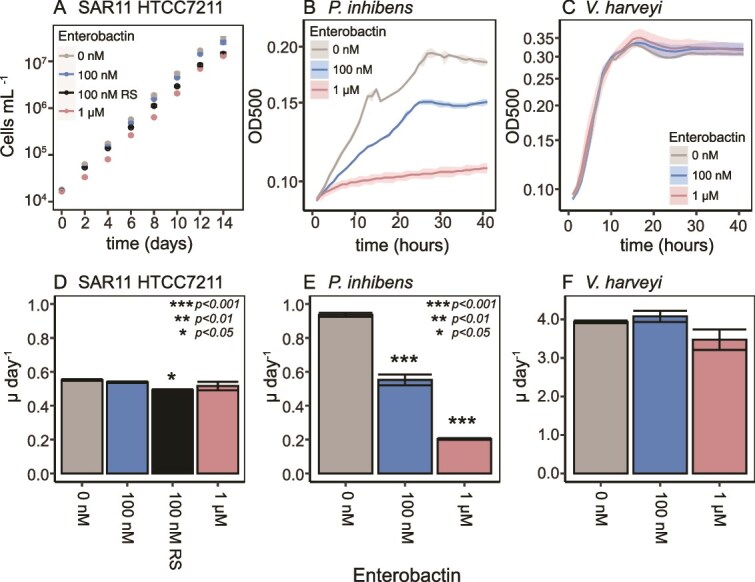
Variable changes in growth rate of SAR11, *P. inhibens,* and *V. harveyi* in the presence of enterobactin. (A) The presence of high or resupplied enterobactin does not lead to substantial decreases in the growth of SAR11 HTCC7211. RS: resupply, enterobactin was added to a final concentration of 100 nM every 2 days throughout the experiment. (B) Growth of *P. inhibens* is severely repressed in the presence of enterobactin. (C) Growth of *V. harveyi* in the presence of enterobactin shows little change from untreated controls. (D) Growth rates for SAR11 calculated using data from days 2–6 in (A). (E) Growth rates for *P. inhibens* calculated using data from 2–8 h in (B). (F) Growth rates for *V. harveyi* calculated using data from 2–8 h in (C). Note the different axes scales. Data shown are the average of biological duplicates ± SD. Statistics shown are from a one-way ANOVA with Tukey post hoc correction and denote differences between the control and the indicated treatment. See Fig. S3 for SAR11 growth in additional siderophore conditions, Fig. S4 for additional *V. harveyi* and *P. inhibens* growth data, and Table S1 for growth rates in additional conditions.

While these models strongly support our growth results, it is notable that apo-enterobactin has been shown to degrade at very high pH [47], which may contribute to its enhanced bioavailability compared to ferrichrome and ferrioxamine. However, when we tested this using LC–MS measurements of apo- and Fe-enterobactin stability at pH 8.1 over 14 days, we found that apo-enterobactin was rapidly degraded over the course of 2 days, but that Fe-enterobactin, which we expect to be the dominant species in our experiments, remained 90% intact after two days, and was still ~50% intact at the end of the experiment (Fig. S2). The evolution of Fe′ was also modeled assuming an apo-enterobactin degradation rate of 0.58 day^−1^, as estimated from the data in Fig. S2. As expected, this increases available Fe′ and suggests sufficiency across all enterobactin treatments (Fig. 4D). Such a result is inconsistent with our growth data where 100 nM enterobactin does lead to mild decreases in growth rates and yields (Table S1) and likely overestimates the contributions of degradation. Further experiments to combat degradation through either resupply or higher concentrations (1 μM) of enterobactin led to some decreases in growth rates and yields (Fig. 5A and D, Table S1) but did not approach those seen with ferrichrome. The decrease in growth on 1 μM enterobactin is notably mild, likely reflecting the abiotic degradation of the large excess of apo-enterobactin (ca. 900 nM for Fe_T_ = 100 nM), as seen in our model (Fig. 4D). Regardless, when compared to experiments with ferrichrome where yields are ~1% of untreated controls even for small siderophore additions (Fig. 2, Table S1), this still represents relatively robust growth. Overall, these modeling and growth experiments confirm that while abiotic degradation of enterobactin may contribute to our phenotypes, differences in the siderophore-iron chemistries are the primary drivers of our results.

### The marine heterotrophs *Vibrio harveyi* and *Phaeobacter inhibens* exhibit contrasting responses to enterobactin

While slow-growing organisms like SAR11 may be able to support themselves by relying on the dissociation of enterobactin-bound Fe, faster-growing organisms may need to access siderophore-bound Fe via cellular uptake machinery in order to meet their growth requirements. As a simple exploration of this idea, we challenged two fast-growing marine heterotrophs, *P. inhibens* and *V. harveyi*, with enterobactin. In the absence of added siderophores, *P. inhibens* growth rates were close to 1 day^−1^ while *V. harveyi* exhibited growth rates of ~4 day^−1^ (Fig. 5, Table S1); growth rates that are ~2–8 times faster than the ~0.5 day^−1^ rate observed in SAR11 cultures. In the presence of 100 nM or 1 μM enterobactin, *P. inhibens* growth was severely inhibited (Fig. 5B and E, Table S1) while *V. harveyi* growth showed little change (Fig. 5C and F, Table S1). The very strong inhibition of *P. inhibens* (especially compared to SAR11 HTCC7211) by 1 μM enterobactin may be due to the fact that *P. inhibens* experiments are short and do not allow for much degradation of the apo-enterobactin. However, these trends were mostly maintained when siderophores were aged for 14 days to mimic possible abiotic degradation that may occur during growth of SAR11; experiments with ferrichrome yielded complex results but suggest the two organisms also have different capacities for direct access to this siderophore (Fig. S4).

In our experiments, *P. inhibens* appears to either not possess or not express a transporter for enterobactin and is likely growing on Fe′. The ~50% inhibition of growth rate in this organism is consistent with reliance on an insufficient supply of Fe from siderophore dissociation and is notably a much more drastic decline in growth rate than seen for SAR11 (compare Fig. 5D  vs  5E). In contrast, *V. harveyi* was able to maintain a much faster growth rate in the presence of enterobactin. This result is confusing if growth is assumed to rely entirely on dissociated iron. However, it is likely that in addition to using Fe′, *V. harveyi* is also able to transport enterobactin. Indeed, this organism produces the siderophore amphi-enterobactin, which bears high structural similarity to enterobactin [48]. Hence, *V. harveyi* may leverage amphi-enterobactin transporters for uptake of enterobactin. Copiotrophic marine bacteria like *P. inhibens* and *V. harveyi* often possess numerous redundant biochemical systems for accessing iron [23, 49], and more detailed experiments would be needed to determine the mechanism employed here. For example, these organisms produce their own siderophores, which may compete with enterobactin [48, 50]. Nonetheless, these proof-of-concept experiments show that purely kinetic-based access to enterobactin-bound Fe likely cannot support fast growth rates and that this type of growth necessitates the use of biochemical uptake mechanisms.

### Implications for the role of siderophores in microbial ecology and marine iron cycling

**Figure 6:**
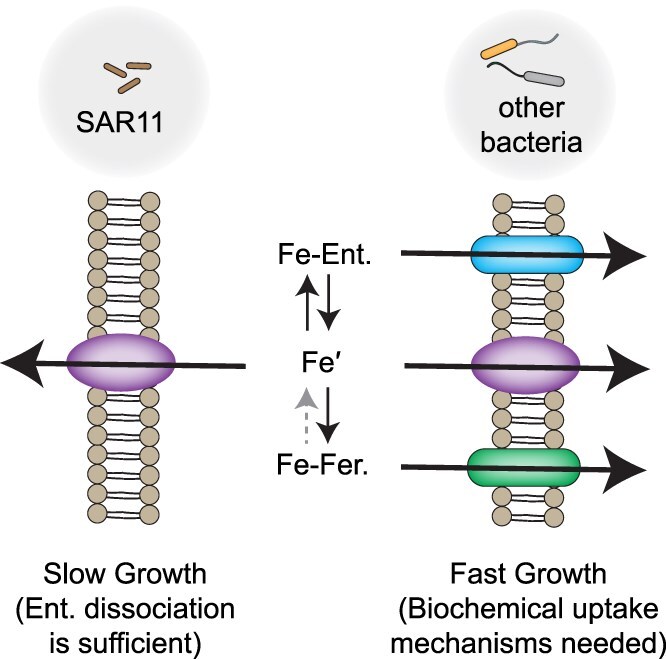
Marine microbes access siderophore-bound iron through different mechanisms. For small, slow growing organisms such as SAR11, the relatively fast dissociation kinetics of enterobactin (but not ferrichrome or ferrioxamine) may provide sufficient iron. For other organisms with faster growth rates, bigger size, and higher iron demands, access to siderophore-bound iron may depend on the more classic mechanisms of uptake via specialized chemical machinery.

It is now well established that marine microbes utilize siderophores to access iron [5, 6, 10, 11, 13, 20, 22, 29–31]. However, it is still unclear how this strategy used by some, but not all marine organisms, affects the cycling and overall bioavailability of iron in marine ecosystems. Our results provide evidence for one solution to this puzzle: access to siderophore-bound Fe might not always be restricted to microbes with specialized uptake machinery. While it has long been the *de facto* assumption that slow-growing marine organisms like SAR11 must depend on dissociated iron [23], there have been few direct demonstrations. A novel biochemical mechanism that allows access to catechol- but not hydroxamate-bound Fe could still be discovered in SAR11—such a strategy might rely, for example, on extracellular degradation via a nonspecific esterase. However, our results can also be explained via kinetics (Fig. 4A–C) without invoking further biochemical mechanisms. In this scenario, which seems likely, our findings should extend to other marine bacteria, helping to explain previous work showing that enterobactin-bound Fe is surprisingly bioavailable to numerous marine microbes [29–31]. While some of these microbes may possess transporters for Fe-enterobactin or its derivatives, others may rely on the same nonspecific kinetic mechanisms seen in our experiments (Fig. 6). Such mechanisms may also allow for nonsiderophore based iron uptake: dissolved organic matter complexes large amounts of marine Fe and labile catechol or other binding groups in this pool may provide a sufficient supply of Fe for slow growers like SAR11.

Our results also contextualize observed differences between hydroxamate and catechol siderophore distributions that are beginning to emerge from field data. We show that iron bound to the catechol enterobactin is highly labile and hence bioavailable, whereas the hydroxamates ferrichrome and ferrioxamine are not, implying that the former may either not persist in the oceans and/or may be disfavored by marine siderophore producers due to its more limited stability and iron binding capacity. Consistent with this, numerous hydroxamates (amphibactin, coelichelin, coprogen, ferrioxamine [8, 10, 11, 13, 51]) and mixed carboxylate/hydroxamates (piscibactin, synechobactin [13, 51]) have now been detected in seawater. In contrast, catechol functionalities are only represented by two structures: the mixed carboxylate/catecholate siderophores petrobactin and alterobactin [13, 52]. This pattern also seems to be reflected in the types of siderophores made by cultured marine organisms, where hydroxamates are more common [6], although these studies are subject to the inherent bias of culture collections. Notably, surveys of the biosynthetic capacity of field populations of marine microbes do detect gene clusters for catecholate siderophores [13]. The failure to detect these siderophores in the oceans may be because they are only produced in specific circumstances or simply don’t persist as long.

Seawater-specific stability and dissociation constants are only available for a limited set of siderophores, and hence, the relatively high bioavailability of Fe-enterobactin and chemical lability of apo-enterobactin (Fig. S2) may be a peculiarity of this catechol siderophore. The mixed functionality siderophore alterobactin is one of the few other siderophores for which *k_d_^FeY^* values have been reported and provides an interesting example as these values are ~6.1 × 10^−4^ h^−1^ and 9 × 10^−4^ h^−1^, for alterobactin A and B, respectively, putting them on par with hydroxamates [33] and suggesting a possible reason why these mixed functionality siderophores might be favored. Our work also relies on experimentally tractable siderophores that are not obviously relevant to marine systems: ferrichrome is a fungal siderophore [53, 54]; enterobactin is made by enterics, and neither has been detected in seawater. However, ferrioxamine, which we find induces phenotypes similar to ferrichrome (Fig. 4E), is made by marine organisms [55, 56] and detected widely in the oceans [8, 10, 11, 13, 57] while amphi-enterobactin, which differs from enterobactin only in the presence of a fatty acid tail and is made by marine *Vibrios* [22, 48] but has not yet been detected in the oceans. If the properties seen in enterobactin (and most likely amphi-enterobactin) are maintained in other marine catechols, it suggests a model in which catechol siderophores serve as a rapidly cycled and more chemically labile pool of iron, whereas hydroxamates form long-lived complexes [58] that supply iron to specific groups of organisms or simply stabilize the dissolved iron pool.

Further studies will be needed to validate these ideas in the field. Nonetheless, our pure culture experiments agree remarkably well with available environmental data and offer testable hypothesis about the different roles of siderophores and microbial uptake strategies in marine microbial ecology and iron cycling. Overall, our work shows that despite the absence of canonical uptake mechanism for siderophores, SAR11 grows robustly in the presence of enterobactin, one of the best studied and strongest known siderophores. This is best explained by the relatively weak iron-binding capacity of enterobactin in seawater and suggests a model of siderophore use in the oceans that includes nonspecific access through kinetics.

## Acknowledgements

We thank the Ward (Princeton) and Chisholm (MIT) labs for providing access to their flow cytometers and M. Saito (WHOI) for assistance with ICP-MS measurements. We are grateful to McRose lab members for helpful comments on the work and in particular to E. Schutt and D. Giacalone for assistance with sampling and flow cytometry. We thank S. Morel for initial MATLAB modeling.

## Supplementary Material

Supplementary_materials_ycag113

Supplementary_materials_ycag113_TableS1

Supplementary_materials_ycag113_TableS2

## Data Availability

All data generated or analyzed during this study are included in this published article and its supplementary information files. All scripts used in the kinetic model area are publicly available here: https://doi.org/10.5281/zenodo.19264293 [60].

## References

[1] Martin JH, Coale KH, Johnson KS. et al. Testing the iron hypothesis in ecosystems of the equatorial Pacific Ocean. *Nature* 1994;371:123–9. 10.1038/371123a0

[2] Boyd PW, Jickells T, Law CS. et al. Mesoscale iron enrichment experiments 1993-2005: synthesis and future directions. *Science* 2007;315:612–7. 10.1126/science.1131669 17272712

[3] Martin JH, Gordon M, Fitzwater SE. The case for iron. *Limnol Oceanogr* 1991;36:1793–802. 10.4319/lo.1991.36.8.1793

[4] Hider RC, Kong X. Chemistry and biology of siderophores. *Nat Prod Rep* 2010;27:637–57. 10.1039/b906679a 20376388

[5] Sandy M, Butler A. Microbial iron acquisition: marine and terrestrial siderophores. *Chem Rev* 2009;109:4580–95. 10.1021/cr9002787 19772347 PMC2761978

[6] Vraspir JM, Butler A. Chemistry of marine ligands and siderophores. *Ann Rev Mar Sci* 2009;1:43–63. 10.1146/annurev.marine.010908.163712 PMC3065440 21141029

[7] Moffett JW, Boiteau RM. Metal organic complexation in seawater: historical background and future directions. *Ann Rev Mar Sci* 2023;16:577–99. 10.1146/annurev-marine-033023-083652 37722713

[8] Mawji E, Gledhill M, Milton JA. et al. Hydroxamate siderophores: occurrence and importance in the Atlantic Ocean. *Environ Sci Technol* 2008;42:8675–80. 10.1021/es801884r 19192780

[9] Velasquez I, Nunn BL, Ibisanmi E. et al. Detection of hydroxamate siderophores in coastal and Sub-Antarctic waters off the South Eastern Coast of New Zealand. *Mar Chem* 2011;126:97–107. 10.1016/j.marchem.2011.04.003

[10] Boiteau RM, Mende DR, Hawco NJ. et al. Siderophore-based microbial adaptations to iron scarcity across the eastern Pacific Ocean. *Proc Natl Acad Sci U S A* 2016;113:14237–42. 10.1073/pnas.1608594113 27911777 PMC5167167

[11] Park J, Durham BP, Key RS. et al. Siderophore production and utilization by marine bacteria in the North Pacific Ocean. *Limnol Oceanogr* 2023;68:1636–53. 10.1002/lno.12373

[12] Li J, Babcock-Adams L, Boiteau RM. et al. Microbial iron limitation in the ocean’s twilight zone. *Nature* 2024;633:823–7. 10.1038/s41586-024-07905-z 39322731

[13] Bundy RM, Manck LE, Repeta DJ. et al. Patterns of siderophore production and utilization at station ALOHA from the surface to mesopelagic waters. *Limnol Oceanogr* 2025;70:128–45. 10.1002/lno.12746

[14] Morris RM, Rappé MS, Connon SA. et al. SAR11 clade dominates ocean surface bacterioplankton communities. *Nature* 2002;420:806–10. 10.1038/nature01240 12490947

[15] Hogle SL, Hackl T, Bundy RM. et al. Siderophores as an iron source for picocyanobacteria in deep chlorophyll maximum layers of the oligotrophic ocean. *ISME J* 2022;16:1636–46. 10.1038/s41396-022-01215-w 35241788 PMC9122953

[16] Lin H, Fischbach MA, Liu D. et al. In vitro characterization of salmochelin and enterobactin trilactone hydrolases IroD, IroE and Fes. *J Am Chem Soc* 2005;127:11075–84. 10.1021/ja0522027 16076215 PMC2536649

[17] Chu BC, Garcia-Herrero A, Johanson TH. et al. Siderophore uptake in bacteria and the battle for iron with the host; a bird’s eye view. *BioMetals* 2010;23:601–11. 10.1007/s10534-010-9361-x 20596754

[18] Schalk IJ . Bacterial siderophores: diversity, uptake pathways and applications. *Nat Rev Microbiol* 2025;23:24–40. 10.1038/s41579-024-01090-6 39251840

[19] Granger J, Price NM. The importance of siderophores in iron nutrition of heterotrophic marine bacteria. *Limnol Oceanogr* 1999;44:541–55. 10.4319/lo.1999.44.3.0541

[20] Cordero OX, Ventouras L-A, DeLong EF. et al. Public good dynamics drive evolution of iron acquisition strategies in natural bacterioplankton populations. *Proc Natl Acad Sci U S A* 2012;109:20059–64. 10.1073/pnas.1213344109 23169633 PMC3523850

[21] D’Onofrio A. et al. Siderophores from neighboring organisms promote the growth of uncultured bacteria. *Chem Biol* 2010;17:254–64. 10.1016/j.chembiol.2010.02.010 20338517 PMC2895992

[22] McRose DL, Baars O, Seyedsayamdost MR. et al. Quorum sensing and iron regulate a two-for-one siderophore gene cluster in *Vibrio harveyi*. *Proc Natl Acad Sci U S A* 2018;115:7581–201807586. 10.1073/pnas.1805791115 29954861 PMC6055174

[23] Hogle SL, Thrash JC, Dupont CL. et al. Trace metal acquisition by marine heterotrophic bacterioplankton with contrasting trophic strategies. *Appl Environ Microbiol* 2016;82:1613–24. 10.1128/AEM.03128-15 26729720 PMC4771312

[24] Hopkinson BM, Barbeau KA. Iron transporters in marine prokaryotic genomes and metagenomes. *Environ Microbiol* 2012;14:114–28. 10.1111/j.1462-2920.2011.02539.x 21883791

[25] Boiteau RM, Repeta DJ. An extended siderophore suite from *Synechococcus* sp. PCC 7002 revealed by LC-ICPMS-ESIMS. *Metallomics* 2015;7:877–84. 10.1039/C5MT00005J 25786191

[26] Hopkinson BM, Morel FMM. The role of siderophores in iron acquisition by photosynthetic marine microorganisms. *BioMetals* 2009;22:659–69. 10.1007/s10534-009-9235-2 19343508

[27] Basu S, Gledhill M, Beer D. et al. Colonies of marine cyanobacteria *Trichodesmium* interact with associated bacteria to acquire iron from dust. *Commun Biol* 2019;2:284. 10.1038/s42003-019-0534-z 31396564 PMC6677733

[28] Kazamia E, Sutak R, Paz-Yepes J. et al. Endocytosis-mediated siderophore uptake as a strategy for Fe acquisition in diatoms. *Sci Adv* 2018;4:eaar4536. 10.1126/sciadv.aar4536 29774236 PMC5955625

[29] Hutchins DA, Witter AE, Butler A. et al. Competition among marine phytoplankton for different chelated iron species. *Nature* 1999;400:858–61. 10.1038/23680

[30] Strzepek RF, Maldonado MT, Hunter KA. et al. Adaptive strategies by Southern Ocean phytoplankton to lessen iron limitation: uptake of organically complexed iron and reduced cellular iron requirements. *Limnol Oceanogr* 2011;56:1983–2002. 10.4319/lo.2011.56.6.1983

[31] Kustka AB, Jones BM, Hatta M. et al. The influence of iron and siderophores on eukaryotic phytoplankton growth rates and community composition in the Ross Sea. *Mar Chem* 2015;173:195–207. 10.1016/j.marchem.2014.12.002

[32] Loomis LD, Raymond KN. Solution equilibria of enterobactin and metal-enterobactin complexes. *Inorg Chem* 1991;30:906–11. 10.1021/ic00005a008

[33] Witter AE, Hutchins DA, Butler A. et al. Determination of conditional stability constants and kinetic constants for strong model Fe-binding ligands in seawater. *Mar Chem* 2000;69:1–17. 10.1016/S0304-4203(99)00087-0

[34] Wu J, Iii GWL. Complexation of Fe(III) by natural organic ligands in the Northwest Atlantic Ocean by a competitive ligand equilibration method and a kinetic approach. *Mar Chem* 1995;50:159–77. 10.1016/0304-4203(95)00033-N

[35] Carini P, Steindler L, Beszteri S. et al. Nutrient requirements for growth of the extreme oligotroph ‘*Candidatus* Pelagibacter ubique’ HTCC1062 on a defined medium. *ISME J* 2013;7:592–602. 10.1038/ismej.2012.122 23096402 PMC3578571

[36] Sunda WG, Price NM, Morel FMM. Trace metal ion buffers and their use in culture studies. In: Andersen R.A. (ed.), Algal Culturing Techniques. Elsevier Academic Press, Burlington MA, USA, 2005, 35–63.

[37] Martocello DE, Morel FMM, McRose DL. H-Aquil: a chemically defined cell culture medium for trace metal studies in *Vibrios* and other marine heterotrophic bacteria. *BioMetals* 2019;32:819–28. 10.1007/s10534-019-00215-2 31542845

[38] Livak KJ, Schmittgen TD. Analysis of relative gene expression data using real-time quantitative PCR and the 2^−ΔΔC^T method. *Methods* 2001;25:402–8. 10.1006/meth.2001.1262 11846609

[39] Tang D, Morel FMM. Distinguishing between cellular and Fe-oxide-associated trace elements in phytoplankton. *Mar Chem* 2006;98:18–30. 10.1016/j.marchem.2005.06.003

[40] Byrne GD, Hindmarsh AC. A polyalgorithm for the numerical solution of ordinary differential equations. *ACM Trans Math Softw* 1975;1:71–96. 10.1145/355626.355636

[41] R: A Language and Environment for Statistical Computing. R Foundation for Statistical Computing, 2025.

[42] Smith DP, Kitner JB, Norbeck AD. et al. Transcriptional and translational regulatory responses to iron limitation in the globally distributed marine bacterium *Candidatus* Pelagibacter ubique. *PloS One* 2010;5:e10487. 10.1371/journal.pone.0010487 20463970 PMC2864753

[43] Lis H, Shaked Y, Kranzler C. et al. Iron bioavailability to phytoplankton: an empirical approach. *ISME J* 2015;9:1003–13. 10.1038/ismej.2014.199 25350155 PMC4817705

[44] Rappé MS, Connon SA, Vergin KL. et al. Cultivation of the ubiquitous SAR11 marine bacterioplankton clade. *Nature* 2002;418:630–3. 10.1038/nature00917 12167859

[45] Giovannoni SJ . SAR11 bacteria: the most abundant plankton in the oceans. *Ann Rev Mar Sci* 2014;9:231–55. 10.1146/annurev-marine-010814-015934 27687974

[46] Hering JG, Morel FMM. Slow coordination reactions in seawater. *Geochim Cosmochim Acta* 2002;53:611–8. 10.1016/0016-7037(89)90004-5

[47] Harris WR, Carrano CJ, Cooper SR. et al. Coordination chemistry of microbial iron transport compounds. 19. Stability constants and electrochemical behavior of ferric enterobactin and model complexes. *J Am Chem Soc* 1979;101:6097–104. 10.1021/ja00514a037

[48] Zane HK, Naka H, Rosconi F. et al. Biosynthesis of amphi-enterobactin siderophores by *Vibrio harveyi* BAA-1116: identification of a bifunctional nonribosomal peptide synthetase condensation domain. *J Am Chem Soc* 2014;136:5615–8. 10.1021/ja5019942 24701966

[49] Hogle SL, Brahamsha B, Barbeau KA. Direct heme uptake by phytoplankton-associated *Roseobacter* bacteria. *mSystems* 2017;2:e00124–16. 10.1128/mSystems.00124-16 28083564 PMC5225302

[50] Wang R, Gallant É, Wilson MZ. et al. Algal p-coumaric acid induces oxidative stress and siderophore biosynthesis in the bacterial symbiont *Phaeobacter inhibens*. *Cell Chem Biol* 2022;29:670–679.e5. 10.1016/j.chembiol.2021.08.002 34437838 PMC8866538

[51] Boiteau RM, Till CP, Coale TH. et al. Patterns of iron and siderophore distributions across the California current system. *Limnol Oceanogr* 2019;64:376–89. 10.1002/lno.11046

[52] Manck LE, Park J, Tully BJ. et al. Petrobactin, a siderophore produced by *Alteromonas*, mediates community iron acquisition in the global ocean. *ISME J* 2022;16:358–69. 10.1038/s41396-021-01065-y 34341506 PMC8776838

[53] Neilands JB . A crystalline organo-iron pigment from a rust fungus (*Ustilago sphaerogena*). *J Am Chem Soc* 1952;74:4846–7. 10.1021/ja01139a033

[54] Emery T, Neilands JB. Structure of the ferrichrome compounds. *J Am Chem Soc* 1961;83:1626–8. 10.1021/ja01468a020

[55] Amin SA, Green DH, Waheeb DA. et al. Iron transport in the genus *Marinobacter*. *BioMetals* 2012;25:135–47. 10.1007/s10534-011-9491-9 21894542

[56] Roberts AA, Schultz AW, Kersten RD. et al. Iron acquisition in the marine actinomycete genus *Salinispora* is controlled by the desferrioxamine family of siderophores. *FEMS Microbiol Lett* 2012;335:95–103. 10.1111/j.1574-6968.2012.02641.x 22812504 PMC4209017

[57] Bundy RM, Boiteau RM, McLean C. et al. Distinct siderophores contribute to iron cycling in the mesopelagic at station ALOHA. *Front Mar Sci* 2018;5:61. 10.3389/fmars.2018.00061

[58] Boiteau RM, Repeta DJ. Slow kinetics of iron binding to marine ligands in seawater measured by isotope exchange liquid chromatography–inductively coupled plasma mass spectrometry. *Environ Sci Technol* 2022;56:3770–9. 10.1021/acs.est.1c06922 35213147

[59] Hudson RJM, Covault DT, Morel FMM. Investigations of iron coordination and redox reactions in seawater using ^59^Fe radiometry and ion-pair solvent extraction of amphiphilic iron complexes. *Mar Chem* 1992;38:209–35. 10.1016/0304-4203(92)90035-9

[60] Associated python code to McRose et al. manuscript submitted to ISME Communications in 2025 (Version 1.0.2) 2026.

