# Supplementary material for: Contrasting bioavailability of enterobactin- and ferrichrome-bound iron to SAR11 and other marine heterotrophs
Source: ISME Commun. 2026 Apr 23;6(1):ycag113. doi: 10.1093/ismeco/ycag113 (PMC13271415; doi:10.1093/ismeco/ycag113)
Supplement: Supplementary_materials_ycag113 [file supplementary_materials_ycag113.pdf]

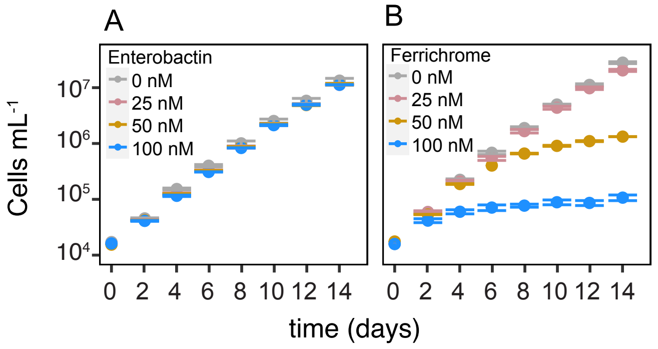

Figure S1. SAR11 shows robust growth in the presence of enterobactin but not ferrichrome. Replicate experiment with SAR11 HTCC1062 showing results similar to those in Fig. 2 for growth in response to enterobactin (A) or ferrichome (B). Data shown are biological duplicates ± SD. The 25 nM ferrichrome treatment at 6 hours is represented by only one replicate. 0 nM ferrichrome and 0 nM enterobactin data are from distinct growth experiments.

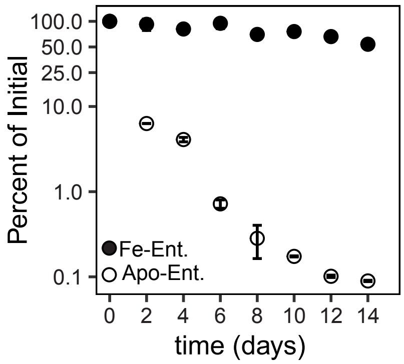

Figure S2. Abiotic degradation of apo-enterobactin (open symbols) is rapid at pH 8.1 but Fe-enterobactin (closed symbols) which is expected to be the primary species in our experimental conditions, is relatively stable. Data shown are for duplicate incubations ± SD.

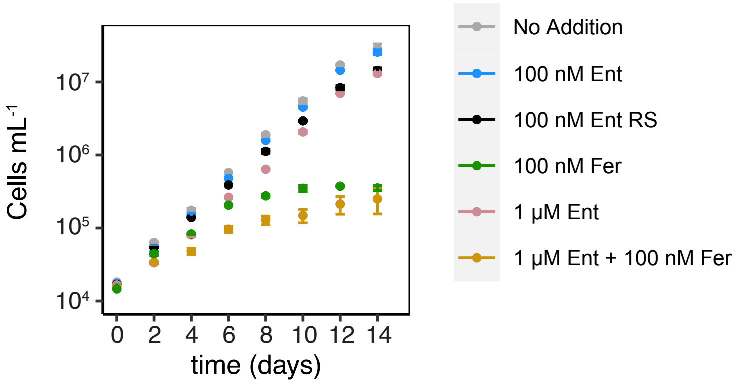

Figure S3. Complete SAR11 HTCC7211 growth data for enterobactin re-supply and mixed enterobactin treatments. Data shown are the same as in Fig. 4E with additional treatments. Data shown are the average of biological duplicates ± SD. Ent:enterobactin, Fer:ferrichrome, Ent RS:resupply, enterobactin was added to a final concentration of 100 nM every 2 days throughout the experiment.

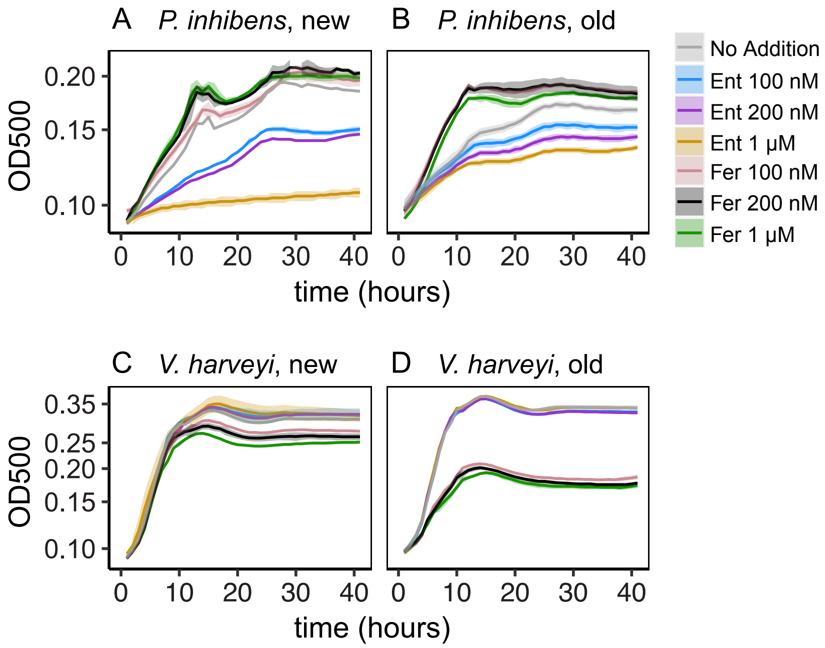

Figure S4. Growth of the marine heterotrophs *P. inhibens* and *V. harveyi* in the presence of siderophores suggests growth on dissociated iron as well as uptake via specialized uptake machinery. Response of *P. inhibens* (A,B) and *V. harveyi* (C,D) to fresh eneterobactin and ferrichrome (A, C) or enterobactin and ferrichrome allowed to age for 14 days in seawater medium (B, D). Data shown are the average of biological duplicates ± SD. Data are the same as shown in Fig. 5 with the addition of data for 200 nM enterobactin and ferrichrome. Ent:enterobactin. Fer:ferrichrome.
