# Supplementary material for: Contrasting bioavailability of enterobactin- and ferrichrome-bound iron to SAR11 and other marine heterotrophs
Source: ISME Commun. 2026 Apr 23;6(1):ycag113. doi: 10.1093/ismeco/ycag113 (PMC13271415; doi:10.1093/ismeco/ycag113)
Supplement: Supplementary_materials_ycag113_TableS2 [file supplementary_materials_ycag113_tables2.docx]

| Primer Name | Sequence 5′ to 3′ | Amplicon (bp) | Source |
| --- | --- | --- | --- |
| *recA* F | AAGCTGGAGGAATTTGTGCG | 197 | This study |
| *recA* R | CGTTAGTGCTGCAACTGAGT | 197 | This study |
| *rpoD* F | TCACAAAGCCCAATGACTGC | 184 | This study |
| *rpoD* R | CACCCTCACCAGATCCTTCA | 184 | This study |
| *sfuA* F | TCTGACGTCCAATTGTTACTTCA | 166 | This study |
| *sfuA* R | GGATGTGATGCGAGTGAACA | 166 | This study |
| *sfuB* F | TTTATCTTTTGCAGTGCCAGCT | 150 | This study |
| *sfuB* R | GCTCCTGCCATACCATCGAA | 150 | This study |

Table S2. RT-qPCR primers used in this study. All primers target genes in HTCC7211.
